# The recognition and expectations of ex-inpatients of mental health services: A web-based questionnaire survey in Japan

**DOI:** 10.1371/journal.pone.0197639

**Published:** 2018-10-15

**Authors:** Akihiro Shiina, Yasutaka Ojio, Aiko Sato, Naoya Sugiyama, Masaomi Iyo, Chiyo Fujii

**Affiliations:** 1 Division of Medical Treatment and Rehabilitation, Chiba University Center for Forensic Mental Health, Chiba-shi, Chiba, Japan; 2 Department of Psychiatry, Chiba University Hospital, Chiba-shi, Chiba, Japan; 3 National Institute of Mental Health, National Center of Neurology and Psychiatry, Kodaira-shi, Tokyo, Japan; 4 Numazu Chuo Hospital, Numazu-shi, Shizuoka, Japan; New York City Department of Health and Mental Hygiene, UNITED STATES

## Abstract

Concern about mental health issues and the treatment of mentally disordered offenders attracts considerable public attention. This study aimed to gather the experiences and opinions of people who have experienced admission to a psychiatric ward in order to grasp their reaction to, and understanding of, the legislation behind the involuntary admission of psychiatric patients. A web-based questionnaire survey was conducted with a total of 379 participants, using a cross-sectional, exploratory design. The data were analyzed using a chi-squared test, Fisher’s exact test, and a logistic regression analysis. According to the results, many patients were satisfied with their treatment during psychiatric admission; however, only few participants said that they had been given an adequate explanation for their involuntary treatment. Most participants expected qualified assistance after discharge, although the prospect of a regular visit from an official was not entirely supported by the participants. Patient satisfaction was relevant to the discussion of their needs after discharge and in developing a crisis plan during admission. These findings suggest that psychiatric patients accept inpatient treatment as long as they receive an adequate explanation. More qualified care such as relapse prevention would be expected to lead to better satisfaction. For them to welcome regular visits from an official, patients may need more information and discussion.

## Introduction

In recent years, mental health has become one of the most devastating health concerns faced by societies globally, including both mental health professionals and the general public. The World Health Organization (WHO) considers several mental disorders to result in living through a damaging disability-adjusted life years (DALYs) [1]. In many nations, public attention has been caught by the rise of mental disorders and the need to find effective treatments for them [2].

In addition, public attention and the media often focus on mental disorders as a cause of criminal offenses [3]. There have been several cases in which the perpetrator of a serious crime was found to have been suffering from a mental disorder. These incidents often attract public anger towards, and suspicion of, offenders with mental disorders. This trend in public opinion has led to discussion of the need for a new provisions within legal systems for treating offenders with mental health issues, in various nations [4–7].

There are two main legislations regarding treatment of mentally disordered offenders in Japan. Mental Health and Welfare Act (MHWA) was originally established in 1950 as the Mental Hygiene Act. This law regulates general rules of inpatient care for patients with mental disorder, and has been repeatedly amended to address several contemporary issues. On the other hand, the Medical Treatment and Supervision Act (MTSA), enforced in 2005, is specialized for the treatment of mentally disorders patients who committed serious crimes [6].

In Japan, in 2016, a massacre occurred at a residence for disabled people in the city of Sagamihara. The defendant, who was an ex-employee of the residence, was suspected to have broken into the facility to kill 19 residents based on his prejudiced against and hatred of disabled people [8]. He had been hospitalized involuntarily for a couple of weeks by order of the prefectural governor under the MHWA, and was a likely cannabis abuser (cannabis is not dominantly abused in Japan.) This incident ignited a broad public argument about several issues relevant to the current situation in Japan. Following the report published by the special team in charge of examining the incident [9], the government submitted an amendment to the MHWA. The amended bill contained a new scheme for the official follow-up of patients hospitalized by the prefectural governor’s order. Some politicians, as well as scholars, disagreed with the new bill, as they were concerned about the potential risk that patients under supervision would their human rights restricted. The bill was abandoned because of the dissolution of the Diet in 2017, and has not since been resubmitted [10]. Instead, the Ministry of Health, Labour and Welfare published guidelines based on a similar scheme to enhance support for patients with mental disorders after discharge. The guidelines said municipalities should support patients only when they give consent; in addition, the need for respectful explanation of the treatment to the patient was emphasized in these guidelines. People who require such support are not limited to patients who have committed an offence [11, 12].

The corresponding author has been a member of a research team focused on reforming the MHWA and also engaged in the establishment of the guidelines mentioned above. The team members had been concerned about the MHWA for its insufficient guarantee of adequate care and treatment for psychiatric patients—not limited to offenders with a mental disorder, but also general patients. Therefore, we believed that discussion of reform of the MHWA should not be limited to the context of the Sagamihara case but should include improvement of the entire range of mental health activities in Japan [13].

Over the course of a number of team conferences, the need became clear for a survey that gathers the opinion of the target population on this legislation (i.e., offenders with mental disorders) was suggested, partially because of a serious backlash against the bill directed by opposition party members who claimed the bill was against the will of psychiatric patients [10].

There are a limited number of studies shedding light on psychiatric patients’ opinions in Japan, According to NPO Zensei Net, an association of psychiatric service users, psychiatric patients are likely to have metabolic diseases at younger ages compared to general patients, based on the result of a questionnaire survey of service users. Furthermore, the majority of patients indicated that they had not been given an explanation about the risk of relapse of their disorder [14]. Another survey of psychiatric patients revealed that 36% of participants had not discussed the risk of relapse with their psychiatrist [15]. These results suggest that psychiatric patients perceive insufficient relapse prevention because of inadequate communication with medical practitioners.

The amended MHWA bill aimed at relapse prevention in patients who had experienced psychiatric admission, by assuring continuity of medical treatment. To achieve this purpose, it is indispensable to meet patient’s needs for services.

Therefore, we believed that gaining an understanding of how people who have experienced admission to a psychiatric ward feel about the current situation of mental health care, and what they want for the future were essential before constructing the new scheme suggested by the bill.

Based on these recommendations, the present study aims to clarify the opinions of Japanese psychiatric patients who have had experiences of psychiatric ward admission about the mental health care system and the care they have received, and to understand whether the kind of mental health care that would be provided based on the reforms in the MHWA would suit their needs and preferences.

## Materials and methods

### Research questions

As mentioned above, we were concerned about the opinions of ex-inpatients with a mental disorder about the mental health care they had received and about a mental health care scheme like the one that would have emerged under the MHWA amendment. How did they regard their treatment in the psychiatric hospital? Were they provided an appropriate explanation? Did they accept the necessity of the treatment? Would the contents of the amended MHWA scheme be welcomed by patients?

To answer to these questions, we conducted this cross-sectional, exploratory, web-based questionnaire survey.

### Participants

To conduct the survey, we made a contract with the Japan Research Center (JRC), a marketing company in Japan, to recruit participants. Cyber Panel (http://www.nrc.co.jp/monitor/cyber201.html), a registration system for web-based questionnaires managed by JRC, was used to search a database consisting of approximately 200,000 people.

We decided to gather the opinions of people registered in Cyber Panel who had been admitted at least once to a psychiatric ward. This was because the new scheme planned in the MHWA amendment mainly targeted psychiatric inpatients who were expected to be discharged.

### Measures

We developed a series of questions based on a discussion conducted by the research group members, with reference to a previous study conducted in Japan [16]. The items from the questionnaire are shown in the S1 Table.

There were five sections in the questionnaire following the screening section whose purpose was to check that each respondent had experience of psychiatric admission.

In Section One, participants were asked about their knowledge and opinion of the MHWA and the MTSA. The content of these questions was copied from that of a previous study in which the corresponding author asked outpatients with mental disorder for their knowledge and opinions about forensic mental health [16]. This section was included in order to compare the present participants’ answers with those in the previous study.

In Section Two, participants were asked about the form of inpatient admission they had experienced (either MHWA or MTSA). In this section, we covered all available forms of psychiatric admission [Involuntary admission by the prefectural governor’s order in the MHWA/ Admission for medical care and protection in the MHWA/ Voluntary admission in the MHWA/ Emergency admission in the MHWA/ Hospitalization order by the court in the MTSA/ Hospitalization for assessment in the MTSA.]

In Section Three, participants were asked about their opinion regarding their latest psychiatric admission. The items in this section were selected according to the judgment of the research team members. In this section, we asked participants to only describe their feeling about the latest admission, specifying which experience was being examined to help them remember their feelings more easily. We also intended to examine how the form of admission influenced their opinion. We hypothesized that involuntarily admitted patients would have lower satisfaction levels.

In Section Four, participants were presented with examples of treatment for aiding inpatients in preparation for discharge and for the prevention of readmission. Participants were asked whether each of these examples was offered to them during their latest psychiatric admission. The aim of this section was to identify the current service level of psychiatric admission.

Finally, in Section Five, we asked whether, if they were to be readmitted to a psychiatric ward, the participants wanted to receive the types of assistance that had been presented to them in the previous section.

### Procedure

The survey was conducted from April 27 to May 31, 2017. JRC sent the questionnaire to all people in the database identified having a medical history of any mental disorders. Only those who had personally experienced psychiatric admission were encouraged to send back the answer form. The respondents were informed of the aim of this study and were told that their personal information would not be sent to us, that it would cost them nothing (except telecommunication costs), that the results would be published, and that participants would be rewarded by JRC. After completion of the survey, JRC anonymized the gathered data and sent them to us.

### Data analysis

We analyzed the data using IBM SPSS Statistics for Windows, Version 24 (IBM Corp., Armonk, NY, United States), and set the level of significance *at p* <0.05. We adopted the chi-squared test, Fisher’s exact test, and logistic regression analysis according to the nature of the variables in question.

### Ethical issues

The study protocol was approved by the ethics committee of the Graduate School of Medicine at Chiba University on April 27, 2017 (no. 189). We did not receive any personal information from the participants or JRC. All respondents were taken to agree to participate if they sent in their answer form. We registered the study with the Clinical Trials Registry of the University Hospital Medical Information Network (UMIN, Tokyo, Japan) with the unique trial number UMIN000027316.

## Results and discussion

### Demographic data

JRC had identified a total of 35,505 people with mental disorders as potential candidates for the survey. The data JRC provided included the following categories of disorders: depression (9,644), sleep disorders (6,082), neuroses (5,189), panic disorder (3,404), bipolar disorder (1,672), bulimia nervosa (1,579), social anxiety disorder (1,378), anorexia nervosa (1,222), obsessive-compulsive disorder (1,005), schizophrenia (925), post-traumatic stress disorder (892), general anxiety disorder (815), attention-deficit hyperactivity disorder (446), and other mental disorders (1,252). JRC sent the request form of the questionnaire to all 35,505 registrars. A total of 379 participants who had at least one experience of admission to a psychiatric ward replied to the questionnaire. All of their data were included in the following analyses. Since there are no exact data regarding how many people in Cyber Panel had experienced a psychiatric admission, the response rate cannot be calculated.

### Section One: Knowledge and opinions about the MHWA and related matters

**Fig 1:**
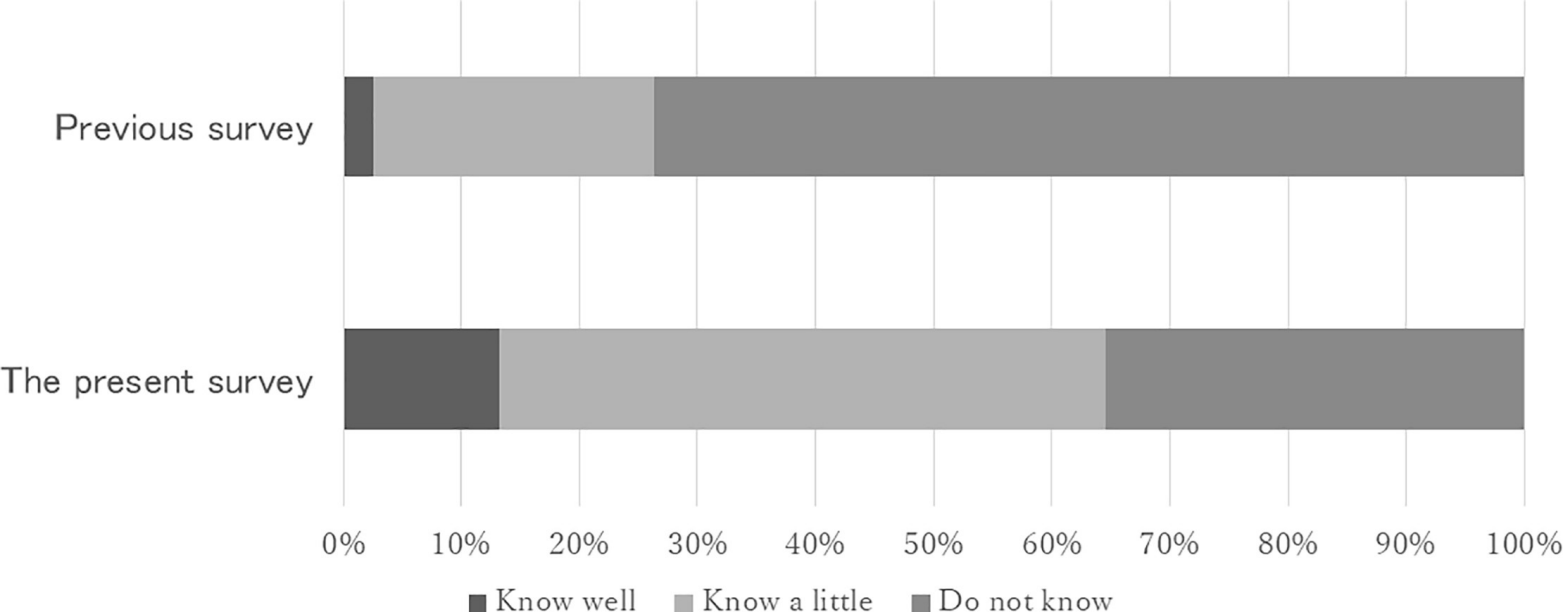
“Do you know the contents of the Mental Health and Welfare Act?” (compared with a previous survey [16]).

A total of 50 (13.2%) participants answered that they knew the MHWA well, while 194 (51.2%) knew it a little, and 134 (35.4%) answered they did not know it. One participant did not answer. A total of 79 (20.8%) knew the fact that the MTSA had come into force in 2005, while 297 (78.4%) did not know it and three did not answer. The proportion who answered that they knew the MHWA well was higher than that of a previous survey conducted by the corresponding author using psychiatric outpatients (chi-squared test, df = 2, chi-squared = 197.43, *p*<0.001. See Fig 1) [16]. With regard to the scheme of involuntary admission by the prefectural governor’s order, 149 (39.3%) were definitely in favor of the policy and 110 (29%) were relatively in favor; 4 (1.1%) were definitely against and 10 (2.6%) were relatively against, and 106 (28%) were neutral. With regard to the MTSA scheme, 159 (42%) were definitely in favor and 92 (24.3%) were relatively in favor; 3 (0.8%) were definitely against and 12 (3.2%) were relatively against; and 106 (28%) were neutral; three did not answer.

### Section Two: Past experience of psychiatric admission

In total, 45 (11.9%) of participants had experienced involuntary admission by the prefectural governor’s order under the MHWA (including urgent involuntary admission, limited to up to 72 hours). In addition, 35 (9.2%) had experienced admission for medical care and protection through the MHWA, 98 (25.9%) had experienced voluntary admission through the MHWA, 11 (2.9%) had experienced emergency admission through the MHWA, 7 (1.8%) had experienced a hospitalization order by a court through the MTSA, and 4 (1.1%) had experienced hospitalization for assessment through the MTSA; 102 (26.9%) participants answered that they had experienced another form of psychiatric admission, while 102 (26.9%) did not know what form of admission they had experienced.

These findings were analyzed by cross-tabulation, which revealed that the participants who had experienced involuntary admission by the prefectural governor’s order had a significantly more unfavorable opinion of this scheme than those without experience of involuntary admission (Fisher’s exact test, df = 4, *p* = 0.034). (See Table 1).

**Table 1:** Opinion of involuntary admission by the prefectural governor’s order (Split by whether or not involuntary admission by the prefectural governor’s order was experienced).

What is your opinion of the scheme of involuntary hospitalization by the prefectural governor’s order for patients at risk of harm to self or others because of mental disorders?					
Involuntary admission by the prefectural governor’s order in the MHWA	Definitely agree	Relatively agree	Neutral	Relatively disagree	Definitely disagree
Experienced	14	8	20	2	1
Did not experience	135	102	86	8	3

### Section Three: Impression of one’s most recent psychiatric admission

Participants’ most recent admissions to a psychiatric ward took the following forms: involuntary admission by the prefectural governor’s order through the MHWA (including urgent involuntary admission) (30, 7.9%), admission for medical care and protection through the MHWA (19/5%), voluntary admission through the MHWA (88, 23.2%), emergency admission through the MHWA (6, 1.6%), hospitalization order by a court through the MTSA (5, 1.3%), hospitalization for assessment through the MTSA (3, 0.8%), and other forms of admission (126, 33.2%). A total of 102 (26.9%) participants did not know the form of their latest admission.

The majority of participants answered that they had accepted the explanation of the necessity of admission. Approximately one-third of participants had felt themselves at risk of harm to self or others at the time of the latest psychiatric admission. Almost one-fifth of participants had received treatment without their consent. Nearly two-thirds of participants believed that their latest admission had been necessary. Among only those who had experienced involuntary admission by the prefectural governor’s order, 19 (67.9%) felt the admission had been necessary. Almost half of the participants answered that they were definitely or relatively satisfied with the treatment they received during their latest admission. These results are shown in Table 2.

**Table 2:** Participants’ views of their most recent psychiatric admission.

Did you accept the necessity of admission and condition of discharge based on fully understanding the explanation?	
Accepted based on understanding the explanation	206 (54.4%)
Well-explained, understood, but did not accept	41 (10.8%)
Did not understand the explanation	16 (4.2%)
Did not receive an explanation	53 (14.0%)
Do not know	62 (16.4%)
At your most recent admission to a psychiatric ward, did you feel you were at risk of harm to self or others because of your mental disorder?	
Yes	122 (32.2%)
No	197 (52.0%)
Do not know	58 (15.3%)
During your most recent admission to a psychiatric ward, did you get any treatment without your consent (e.g. forced injection, restriction of telecommunication, seclusion, and restraint)?	
Yes	75 (19.8%)
No	263 (69.4%)
Do not know	
Do you believe that your most recent admission to a psychiatric ward was necessary for you?	
Yes	248 (65.4%)
No	45 (11.9%)
Uncertain	84 (22.2%)
Were you satisfied with the treatment in your most recent admission?	
Definitely satisfied	89 (23.5%)
Relatively satisfied	100 (26.4%)
Neutral	101 (26.6%)
Relatively unsatisfied	34 (9.0%)
Definitely unsatisfied	52 (13.7%)

### Section Four: Assistance offered in the most recent psychiatric admission

**Fig 2:**
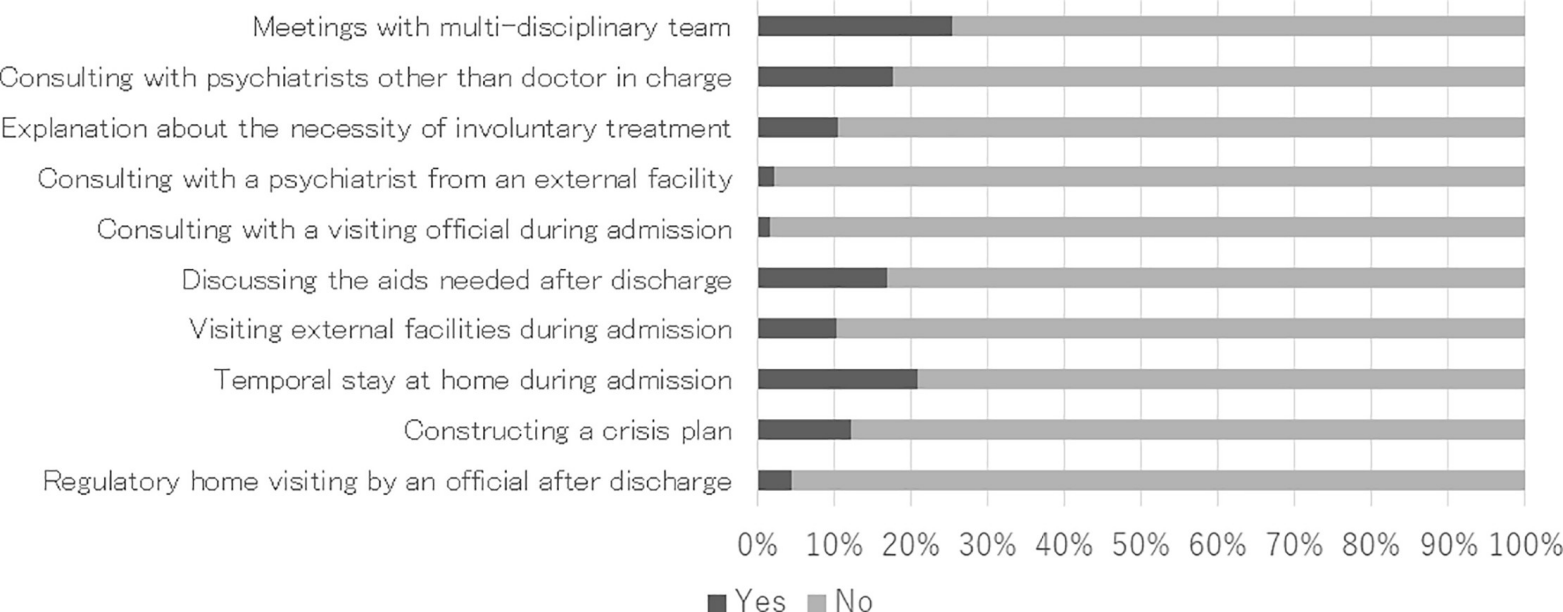
Treatment the participants had received during their most recent admission to a psychiatric ward.

In all ten of the items, only a minority of participants answered that they had been given the types of assistance that were described (see Fig 2). For example, only 40 (10.1%) of the participants answered that they had been given a clear explanation about the necessity of involuntary treatment. The proportion was not significantly different between those who actually received treatment without their consent and those who did not receive any involuntary treatments (chi-squared test, df = 2, chi-squared = 2.496, *p* = 0.299).

For additional statistical analyses, we converted the answers concerning patients’ most recent psychiatric admission satisfaction level into a binary scale: “definitely satisfied” and “relatively satisfied” were converted into “satisfactory,” while “neutral,” “relatively unsatisfied,” and “definitely unsatisfied” were converted into “unsatisfactory.” After the conversion, 189 (49.4%) participants were classified as having had a “satisfactory experience,” and 187 (49.3%) as “unsatisfactory.” Next, we examined whether their satisfaction was altered based on explanation of the necessity of involuntary treatment. We found no significant difference in the proportion of satisfied participants, regardless of the explanation given (chi-squared test, df = 1, chi-squared = 2.854, *p* = 0.091). However, when stratification with the existence of involuntary treatment was applied, among participants who received involuntary treatments, those who had received an explanation of the necessity of involuntary treatment were significantly more likely to be satisfied than those who had not (Fisher’s exact test, df = 1, *p* = 0.016.)

Furthermore, we applied a logistic regression analysis to identify the factors that influenced inpatients’ satisfaction. We set the binary value “satisfaction” above as the dependent variable and input the ten items of treatment for assisting inpatients (from Section 4) as the independent variables. We used stepwise, logistic regression analysis, with increasing variables. As a result, two items: “discussing the assistance needed after discharge” (B = 0.886, SE = 0.317, Wald = 7.819, df = 1, *p* < 0.005, Exp(B) = 2.427) and “constructing a crisis plan” (B = 0.847, SE = 0.375, Wald = 5.107, df = 1, *p* < 0.024, Exp(B) = 2.322), were extracted as significantly relevant to satisfaction level.

### Section Five: Preferred assistance in future psychiatric admissions

**Fig 3:**
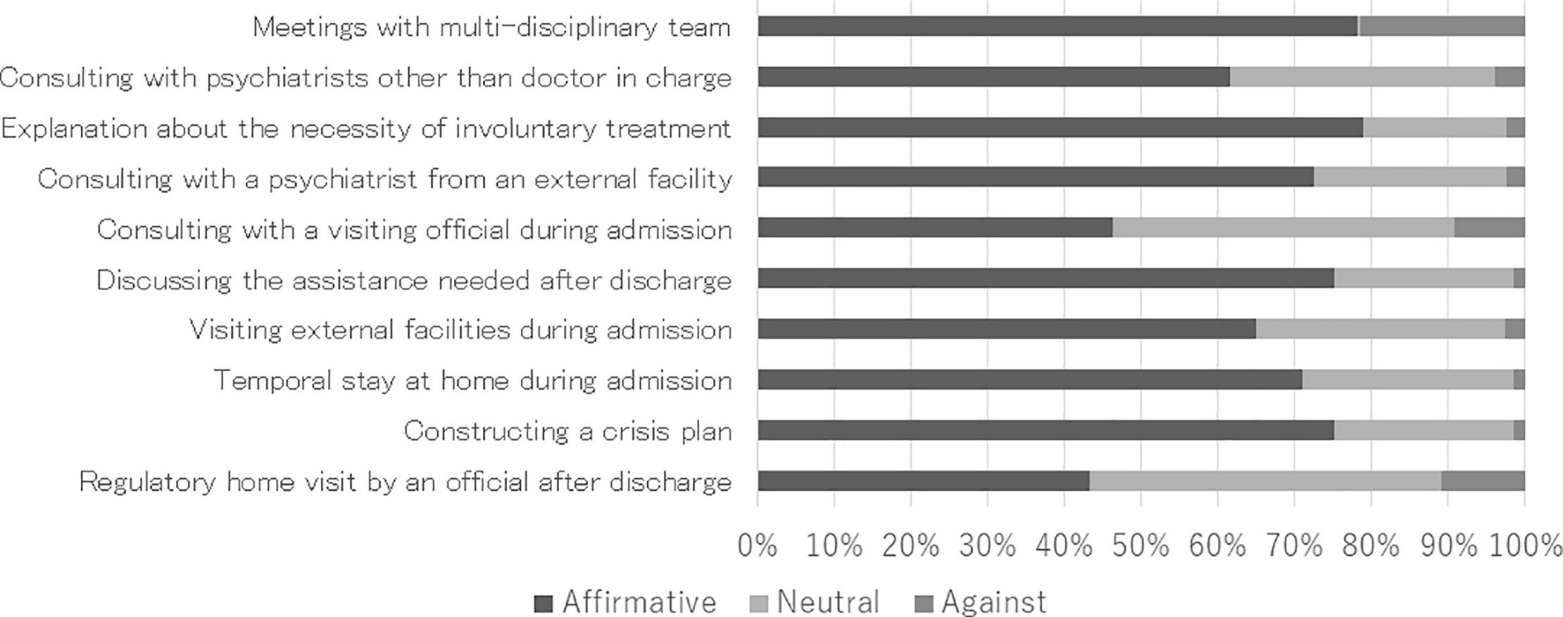
Opinions about assistance preferred in a future admission.

In all ten items, positive opinions were much more prevalent than negative ones. However, for the items “consulting with a visiting official during admission” and “regulatory home visiting by an official after discharge,” a considerable number of participants answered that they were uncertain (See Fig 3).

## Discussion

In the present study, we conducted a questionnaire with people who had experienced admission to a psychiatric ward. This survey focused on patients’ opinion toward mental health care services. The results from this study give us some important information regarding the opinions of these psychiatric patients. Over 35,000 patients (approximately one percent of all psychiatric patients in Japan) [17] were screened to detect more than 300 participants with experiences of psychiatric admission. We are aware of no surveys of a similar scale using psychiatric patients in Japan.

For the present study, we constructed a questionnaire based on discussion among the research team whose members were engaged in the reform of the MHWA. Therefore, the items included in the questionnaire were specific to the planned amendment of the MHWA scheme. We believe this was advantageous for the utilizability of our results to inform further discussion on reforming mental health services in Japan. On the other hand, this feature makes it difficult to generalize the results to psychiatric patients in other nations.

There are some psychometric tools that are standardized for evaluating the satisfaction level and emotional climate of psychiatric inpatients [18,19]. Adopting them would have improved the generalizability of this study. However, we could not find such tools available in Japanese language without burdening respondents. We had to prioritize feasibility and quick completion of this online survey; therefore, we narrowed down the items of the questionnaire.

In this study, a considerable proportion of the participants answered that they had knowledge of the legislation regarding mental health services. Ex-inpatients were more likely to know the MHWA well, compared to general outpatients. These findings suggest that individuals are best able to gain knowledge of the mental health law through their own experience. This estimation is consistent with the fact that the MHWA demands that medical practitioners explain to psychiatric inpatients about their legal status and human rights. On the other hand, it appears that participants did not possess enough knowledge of the circumstances governing their psychiatric admission. A total of 126 (33.2%) participants answered that their latest form of admission to a psychiatric ward was in the category “other forms of admission.” It is, however, extremely rare for a patient to be hospitalized under neither the MHWA nor the MTSA [20]. Thus, it seems that up to a third of participants might have misunderstood the form of admission they experienced, and so it is possible that some patients were not as familiar with mental health law as they believed. Alternatively, some patients may not have been concerned about their legal status at the time of their admission, so may not have paid attention to the information provided to them.

Many participants said at the time of the survey that their latest admission had been necessary. This result is similar to those in studies previously conducted in other nations, such as Switzerland [21] and Ireland [22]. However, the participants whose latest admission was an involuntary admission by the prefectural governor’s order had a relatively negative opinion toward involuntary admission (see Table 1.) This result is consistent with that of a previous study in Japan [16], and means that the scheme of involuntary admission by the prefectural governor’s order may require the fostering of a better level of understanding in its patients to increase their level of acceptance.

An additional analysis revealed that the satisfaction of participants who had received involuntary treatments was associated with a thorough explanation. High satisfaction in patients may motivate them to continue treatment, leading to better outcomes [23]. Thus, in cases where patients are confused about the necessity of the treatment, health practitioners should spend more time explaining it.

Approximately a fifth of participants answered they had received some treatment without their consent. The MHWA permits an administrator of a psychiatric hospital to place some restrictions on an inpatient’s behavior, such as seclusion and restraint, as far as this adheres to appropriate legal procedures (judged by licensed psychiatrists, proper medical recording, regulatory reassessment, and so on.) However, how to address medical treatment without informed consent, such as forced injection, is not described in the MHWA. Wider and more detailed investigation is needed to clarify the current situation of forced treatment in psychiatric hospitals in Japan.

Although we believed before conducting the survey that many psychiatric hospitals were already offering various kinds of assistance for qualified care, most participants answered that they had not been given them. The distribution of answers was not altered by sorting by the form of admission. Among them, the result that only 10.1% of participants believed that they received a proper explanation regarding the necessity of involuntary treatment is surprising. The MHWA requires practitioners to explain the possibility of involuntary treatment to all inpatients at the beginning of admission. In reality, it appears that most patients did not understand the explanation that was provided to them. If there are cases where medical practitioners adopt coercive means to provide treatment without proper explanation to psychiatric inpatients, this is something that needs to be addressed as a matter of priority.

On the other hand, positive opinions were more common than negative ones on every item that was asked in section 5. Overall, the participants seemed happy to be given the kinds of assistance that were planned to be introduced after the amendment of the MHWA. Patient satisfaction was positively associated with discussing the assistance needed after discharge and with constructing a crisis plan. These items can thus be interpreted as helping prevent readmission. Identifying the risk of readmission with providing some solutions in advance to the situation are important, not only for good treatment outcomes but also for better patient satisfaction.

An exception to this is that officials visiting a patient’s home were not entirely welcome. Not many participants actively opposed the proposal, but half withheld their opinion towards receiving an official’s visit after being discharged. It is possible that patients are not willing to be involved in a scheme of official support for discharged patients, or perhaps they have an inaccurate perception of what an official visit would entail.

A major limitation of this study is the sampling bias. First, there may have been people with experience of psychiatric admission who did not reply to our questionnaire. Since neither JRC nor we know how many people registered with JRC had experience of psychiatric admission, the response rate of this survey was uncertain, and is possible some people with experience of psychiatric admission hesitated to express their opinion on this survey. Second, the participants were limited to those with at least one experience of psychiatric admission. Thus, it is possible that their mental disorders were more severe than those in general psychiatric patients, most of whom have never experienced psychiatric admission. On the other hand, the participants had registered with JRC voluntarily, which suggests that they were capable of making an account on an internet service and were willing to respond to some social surveys. Taken together, these facts suggest that this study’s participants were those who had recovered from a relatively severe mental state that needed inpatient care. It may not be the case that the participants are representative of psychiatric patients as a whole; regardless, the results from this study did provide important information regarding the opinions of psychiatric patients, due to the large screening population and sample size.

Finally, we will give suggestions regarding desirable amendment of the MHWA based on this study’s findings. The scheme of involuntary admission itself should be maintained, as many ex-inpatients accepted that it was necessary. However, clear and persuasive explanations to each patient about any intervention should be mandated. Despite being mentioned in the present legislation, stricter regulation is required because many participants seem to receive inadequate explanation and dislike it. Considering that many participants want to be given assistance in the community, a scheme of special care for patients after discharge can be considered. When introducing support services by officials, such as regulatory home visits, advance consultation with the patient will be needed.

## Conclusions

We conducted a national web-based anonymous questionnaire survey to gather data from hundreds of participants with experience of psychiatric admission. Although the majority of them accepted the necessity of involuntary admission, they felt they were not given a proper explanation about it at the latest admission. The contents of the support services planned in the amended MHWA seem to be welcomed by patients. The best way of dealing with an official visiting a patient’s home after discharge should be discussed more concretely.

## Supporting information

S1 Table The items of the questionnaire. (DOCX)
