# Supplementary material for: The recognition and expectations of ex-inpatients of mental health services: A web-based questionnaire survey in Japan
Source: PLoS One. 2018 Oct 15;13(10):e0197639. doi: 10.1371/journal.pone.0197639 (PMC6188626; doi:10.1371/journal.pone.0197639)
Supplement: S1 Table — (DOCX) [file pone.0197639.s001.docx]

# **S1 Table The items of the questionnaire**

### Section 1

(1) Do you know the content of the Mental Health and Welfare Act? [Know well/Know a little/Do not know]

(2) Do you know that the Medical Treatment and Supervision Act came into force in 2005? [Know/Do not know]

(3) What is your opinion toward the scheme of involuntary hospitalization by the prefectural governor’s order for patients at risk of harm self or others due to mental disorders? [Definitely agree/Relatively agree/Neutral/Relatively disagree/Definitely disagree]

(4) What is your opinion toward the scheme of the Medical Treatment and Supervision Act for preventing relapse and reintegration to the society of patients who had committed serious harms due to mental disorders? [Definitely agree/Relatively agree/Neutral/Relatively disagree/Definitely disagree]

### Section 2

Choose all the forms of admission you have experienced in a psychiatric ward (multiple choice admitted). [Involuntary admission by the prefectural governor’s order under the MHWA/ Admission for medical care and protection under the MHWA/ Voluntary admission under the MHWA/ Emergency admission under the MHWA/ Hospitalization order by the court under the MTSA/ Hospitalization for assessment under the MTSA/ Other form of admission to a psychiatric ward/ Form unknown]

### Section 3

(1) What is the latest form of admission to a psychiatric ward that you experienced? [Involuntary admission by the prefectural governor’s order in the MHWA/Admission for medical care and protection in the MHWA/Voluntary admission in the MHWA/Emergency admission in the MHWA/Hospitalization order by the court in the MTSA/Hospitalization for assessment in the MTSA/Other form of admission to a psychiatric ward/Form unknown]

(2) At the latest admission to a psychiatric ward, did you accept the necessity of admission and condition of discharge based on full understanding the explanation? [Accepted based on understanding the explanation/Well-explained, understood, but did not accept/Did not understand the explanation/Did not receive explanation/Do not know]

(3) At the latest admission to a psychiatric ward, did you feel you were at risk of harm to self or others due to your mental disorder? [Yes/No/Do not know]

(4) During the latest admission to a psychiatric ward, did you get any treatment without your consent (e.g. forced injection, restriction of telecommunication, seclusion, and restraint)? [Yes/No/Do not know]

(5) Do you believe that your most recent admission to a psychiatric ward was necessary for you? [Yes/No/Uncertain]

(6) Were you satisfied with the treatment in your most recent admission? [Definitely satisfied/Relatively satisfied/Neutral/Relatively unsatisfied/Definitely unsatisfied]

### Section 4

Choose the content you received during the latest admission to a psychiatric ward (multiple choice admitted).

(1) Meetings with multiple disciplinary teams

(2) Consulting with psychiatrists other than the doctor in charge

(3) Receiving clear explanations about the necessity of involuntary treatment

(4) Consulting with a psychiatrist from an external facility when you do not accept the inpatient treatment

(5) Consulting with a visiting official (e.g. public health nurse) during admission

(6) Discussing the contents of aids estimated to be needed after discharge

(7) Visiting external facilities which would be concerned after discharge (e.g. day care center) during admission

(8) Temporal stay at home during admission

(9) Constructing a crisis plan

(10) Regular visits to the home of an official (e.g. public health nurse) after discharge

### Section 5

Do you agree with each of the ideas below in the event that you have to be readmitted to a psychiatric ward?

(1) Meetings with multiple disciplinary teams [Yes/No/Uncertain]

(2) Consulting with psychiatrists other than the doctor in charge [Yes/No/Uncertain]

(3) Receiving clear explanations about the necessity of involuntary treatment [Yes/No/Uncertain]

(4) Consulting with a psychiatrist from an external facility when you do not accept the inpatient treatment [Yes/No/Uncertain]

(5) Consulting with a visiting official (e.g. public health nurse) during admission [Yes/No/Uncertain]

(6) Discussing the contents of aids estimated to be needed after discharge [Yes/No/Uncertain]

(7) Visiting external facilities which would be concerned after discharge (e.g. day care center) during admission [Yes/No/Uncertain]

(8) Temporal stay at home during admission [Yes/No/Uncertain]

(9) Constructing a crisis plan [Yes/No/Uncertain]

(10) Regular visits to the home of an official (e.g. public health nurse) after discharge [Yes/No/Uncertain]
